# Regulating Root Fungal Community Using *Mortierella alpina* for *Fusarium oxysporum* Resistance in *Panax ginseng*

**DOI:** 10.3389/fmicb.2022.850917

**Published:** 2022-05-12

**Authors:** Yan Wang, Liwei Wang, Meng Suo, Zhijie Qiu, Hao Wu, Min Zhao, Hongyan Yang

**Affiliations:** ^1^College of Life Sciences, Northeast Forestry University, Harbin, China; ^2^Key Laboratory for Enzyme and Enzyme-like Material Engineering of Heilongjiang, Harbin, China

**Keywords:** *Mortierella alpina*, *Panax ginseng*, microbial community, *Fusarium oxysporum*, resistance

## Abstract

Plant-associated microbes play important roles in plant health and disease. *Mortierella* is often found in the plant rhizosphere, and its possible functions are not well known, especially in medical plants. *Mortierella alpina* isolated from ginseng soil was used to investigate its effects on plant disease. The promoting properties and interactions with rhizospheric microorganisms were investigated in a medium. Further, a pot experiment was conducted to explore its effects on ginseng root rot disease. Physicochemical properties, high-throughput sequencing, network co-occurrence, distance-based redundancy analysis (db-RDA), and correlation analysis were used to evaluate their effects on the root rot pathogen. The results showed that *Mortierella alpina* YW25 had a high indoleacetic acid production capacity, and the maximum yield was 141.37 mg/L at 4 days. The growth of *M. alpina* YW25 was inhibited by some probiotics (*Bacillus*, *Streptomyces*, *Brevibacterium*, *Trichoderma*, etc.) and potential pathogens (*Cladosporium*, *Aspergillus*, etc.), but it did not show sensitivity to the soil-borne pathogen *Fusarium oxysporum*. Pot experiments showed that *M. alpina* could significantly alleviate the diseases caused by *F. oxysporum*, and increased the available nitrogen and phosphorus content in rhizosphere soil. In addition, it enhanced the activities of soil sucrase and acid phosphatase. High-throughput results showed that the inoculation of *M. alpina* with *F. oxysporum* changed the microbial community structure of ginseng, stimulated the plant to recruit more plant growth-promoting bacteria, and constructed a more stable microbial network of ginseng root. In this study, we found and proved the potential of *M. alpina* as a biocontrol agent against *F. oxysporum*, providing a new idea for controlling soil-borne diseases of ginseng by regulating rhizosphere microorganisms.

## Introduction

Ginseng (*Panax ginseng* C. A. Meyer), a member of the Araliaceae family, is a valuable medicinal plant with multiple functions, such as enhancing organ functioning, inhibiting inflammation, preventing tumors and diseases, and potentially inhibiting COVID-19 (Han et al., 2018; Li et al., 2020b; Lee and Rhee, 2021). It is estimated that the global ginseng market, including ginseng root and the processed products, is worth $2,084 million (Baeg and So, 2013). Ginseng is cultivated in many countries, such as China, United States, and South Korea. As a perennial plant, ginseng grows in cold and humid environments and is prone to various diseases during growth. *F. oxysporum* can cause many plant diseases, such as tomato wilt, potato dry rot, and soybean root rot (Palmieri et al., 2020; Han et al., 2021; Ren et al., 2021). Root rot is a serious soil-borne disease of ginseng that damages ginseng of all ages and can lead to crop failure in severe cases (Farh et al., 2018). *F. oxysporum* is one of the main pathogens causing root rot in ginseng (Punja et al., 2008).

Currently, ginseng soil-borne diseases are mainly controlled by chemical agents. The frequent use of chemical fungicides has led to many problems, including increased pathogen resistance to fungicides, destruction of the soil microenvironment, high levels of toxic substances in ginseng, and environmental pollution (Wang et al., 2021a). Biological control is an environmentally friendly method of controlling plant diseases using beneficial microorganisms to regulate the microbiological composition of the soil. This effectively protects plants from pathogenic microorganisms while gradually leading to positive microbial community succession. A large number of commercial microbial agents have been developed from endophytic and rhizospheric microorganisms, such as *Bacillus*, *Pseudomonas*, and *Trichoderma* (John et al., 2010; You et al., 2016; De Silva et al., 2019). Different plants have different physiological characteristics and rhizospheric soil microdomains. Consequently, screening microorganisms from native plants and rhizospheric soil can easily enhance the effectiveness of biocides (Bagy et al., 2019; Azabou et al., 2020). Therefore, it can become an effective means to control soil-borne diseases.

*Mortierella*, due to its ability to degrade organic pollutants, can be used for soil remediation. *Mortierella* has also been detected in the rhizosphere and bulk soils of many plants (Liu et al., 2021; Tong et al., 2021; Xiang et al., 2021; Zhou et al., 2021). Some studies have indicated that *Mortierella* is related to soil disease inhibition, and may inhibit the diseases caused by *Fusarium* and participate in the transformation of phosphorus in soil. This is beneficial for soil health and nutrient absorption in plants from soil (Li et al., 2020a; Liu et al., 2020a). However, another study reported that *Mortierella* was a dominant plant pathogen (Guo et al., 2021). This controversy suggests that the effects of *Mortierella* on plants may be species-specific. In addition, the current analysis of *Mortierella* disease inhibition has not reached a consensus on whether *Mortierella* achieves inhibition and growth promotion in pathogenic microorganisms by affecting the bacterial or fungal community in soil or plants (Li et al., 2020a; Guo et al., 2021). Therefore, it is vital to discuss possible plant-specific probiotic effects of microbial species. This will contribute to better defining the scope of action and functions of biocontrol microorganisms.

*Mortierella* has also been detected in the rhizosphere of *Panax ginseng* (Li et al., 2018; Liu et al., 2020b). However, its possible function during *Panax ginseng* cultivation remains unclear. Our previous research showed that *Mortierella* accounted for different proportions of fungal communities under different soil planting conditions, with the highest proportion in forest soil and the lowest in 4-year ginseng-cultivated soil. This indicates that there is a positive correlation between *Mortierella* and the health of ginseng cultivation soil (Wang et al., 2021b). However, it is unclear whether it can be used as a possible biocontrol fungus to improve the resistance of ginseng to soil-borne diseases.

Here, an *M. alpina* strain YW25 isolated from ginseng rhizosphere soil was inoculated into the ginseng rhizosphere to test its possible pathogen resistance and biocontrol potential during *Panax ginseng* cultivation. *F. oxysporum* strain YFW32, which causes ginseng root rot, was used as the pathogen. In this study, we aimed to determine (1) the effects of inoculation of native *M. alpina* on ginseng and rhizosphere soil; (2) whether *M. alpina* has the ability to help plants resist the invasion of pathogens; and (3) if it does, how is the underlying mechanism?

## Materials and Methods

### Microbial Strains

*Mortierella alpina* YW25 was isolated from ginseng rhizosphere soil, and *F. oxysporum* YFW32 was isolated from diseased ginseng roots. The above strain sequences were been stored in DDBJ/EMBL/GenBank using DDBJ quick annotation and submission tool (DFAST),^1^ and their login numbers were LC663965 and LC656545, respectively. The strains were stored at −80°C and then streaked on PDA plates, cultured at 28°C for 7 days, and transferred twice for subsequent tests.

### Analysis of Growth-Promoting Potential of *Mortierella alpina*

The Salkowski colorimetric method (Gordon and Weber, 1951) was used to evaluate the IAA production capacity of *M. alpina* YW25. Briefly, six PDA plugs with *M. alpina* YW25 mycelia (5 mm diameter) were inoculated in flasks containing 100 ml PDB liquid medium and 3 mM tryptophan. The flasks were maintained at 28°C for 2–7 days at 180 rpm. Uninoculated medium was used as the control. Then, 2 ml of culture was centrifuged at 10,000 rpm and 4°C for 10 min. The supernatant was mixed with Salkowski reagent in equal volumes, and the reaction was developed at 25°C in the dark for 30 min. The absorbance was measured at 535 nm. A calibration curve was established for calculating IAA concentration (5–100 mg/L) at 535 nm using pure IAA. The values were averaged over triplicates.

The solubility of *M. alpina* YW25 inorganic phosphorus was evaluated using Pikovskaya’s (PVK) medium (Pikovskaya, 1948). The 1 L medium consisted of 10 g glucose, 0.3 g NaCl, 0.3 g KCl, 0.5 g (NH_4_)_2_SO_4_, 0.3 g MgSO_4_·7H_2_O, 0.03 g MnSO_4_·4H_2_O, 0.03 g FeSO_4_·7H_2_O, 5 g Ca_3_(PO_4_)_2_, 18 g agar, and 1 L distilled water, and was adjusted to pH 7.0–7.2. *M. alpina* YW25 was inoculated into plates containing PVK agar medium. The inoculated plates were incubated in the dark at 28°C for 7 days. Clear halos were observed around the colonies, which indicated that the isolate solubilized inorganic phosphate. Lecithin (P7443, Sigma-Aldrich, United States) was used instead of Ca_3_(PO_4_)_2_ to evaluate its ability to dissolve organophosphorus (Wei et al., 2018). This was carried out by the same process as inorganic phosphorus. The phosphate solubility index (SI), which is the whole diameter zone (diameter of halo + diameter of colony) ÷ colony diameter, was used to evaluate the phosphorus solubility of the strain. The values were averaged over triplicates.

Chrome Azurol S (CAS; Schywn and Nielands, 1987) was used to evaluate the siderophore production capacity of *M. alpina* YW25. PDA plugs with *M. alpina* YW25 mycelia (5 mm diameter) were inoculated on CAS plates and incubated at 28°C for 7 days. The formation of an orange halo around the colony was observed. Larger halos had darker colors, which indicated a higher yield of siderophores. Six PDA plugs with *M. alpina* YW25 mycelia were inoculated in 100 ml of PDA liquid medium. The flasks were maintained at 28°C for 7 days at 180 rpm. Subsequently, 2 ml of culture at 4°C was centrifuged at 10,000 rpm for 10 min. The supernatant was mixed with CAS solution in equal volumes, and the reaction was carried out at 25°C in the dark for 1 h. The absorbance was detected at 630 nm (A), and the uninoculated medium was used as the control (Ar). Siderophores produced by the isolate were measured as percent siderophore units (% SU), and were calculated according to the following formula: % SU = (Ar–A) ÷ Ar × 100. The values were averaged over triplicates (Machuca and Milagres, 2003).

PDA plugs containing *M. alpina* YW25 mycelia were inoculated on PDA plates containing 0.2% soluble starch, 0.5% carboxymethyl cellulose, 0.5% xylan, 1% pectin, and 1% skim milk powder and cultured at 28°C for 7 days to evaluate the activity of amylase, cellulase, xylanase, pectinase, and protease, respectively. The plate containing 0.2% soluble starch was treated with a 1% iodine solution. A transparent halo around the colony indicated amylase activity. Congo red solution (0.2%) was added to the plates containing 0.5% carboxymethyl cellulose and 0.5% xylan. Following this, the plates were washed with 1 M NaCl. Yellow halos were observed around the colonies, which indicated cellulase and xylanase activities, respectively. When 1% cetyl trimethyl ammonium bromide (CTAB) was added to the plate containing 1% pectin, a transparent halo appeared around the colony, which indicated pectinase activity. After the fungi were cultured on PDA plates containing 1% skim milk powder, a transparent hydrolytic halo appeared around the colony, which indicated protease activity (Sopalun and Iamtham, 2020; Liu et al., 2020c; Sopalun et al., 2021).

Six PDA plugs with *M. alpina* YW25 mycelia (5 mm diameter) were inoculated in flasks containing 100 ml YM liquid medium (Papagianni and Moo-Young, 2002). The flasks were maintained at 28°C for 5 days at 180 rpm. Mix 1 ml culture supernatant in equal volume with a phosphate buffer (pH 7.0) containing 1% (w/v) casein, and incubated for 10 min at 30°C. Two milliliter of 0.4 M trichloro acetic (TCA) acid was added to terminate the reaction. The mixture containing the culture supernatant was incubated for 30 min at 25°C followed by centrifugation at 10,000 rpm for 5 min. Five microliter of 0.4 M Na_2_CO_3_ was then mixed with the supernatant (1 ml) and after 10 min, 1 ml of Folin reagent was added to each tube. The tubes were allowed to stand for 30 min at 30°C and then the absorbance was measured at 660 nm. Similar approach was used to prepare the control except casein was added only after the reaction was stopped. 1 U = the amount of enzyme required to liberate one microgram (1 μg/ml) of tyrosine under the assay conditions described (Chimbekujwo et al., 2020). The values were averaged over triplicates.

### *In vitro* Analysis of Interactions Between *Mortierella alpina* and Rhizosphere Microorganisms

The interaction between *M. alpina* YW25 and ginseng rhizosphere microorganisms was evaluated *in vitro* using the plate confrontation method (Cong et al., 2019). The 17 fungi, 15 bacteria, and two actinomycetes used for confrontation were isolated from ginseng rhizosphere soil. *M. alpina* YW25 and rhizosphere fungi were symmetrically and equidistantly inoculated on a PDA plate 2.5 cm away from the center, and cultured at 28°C in the dark for 7 days. *M. alpina* YW25 was placed in the center of LB and Gao’s No.1 plates, and bacterial and actinomycete colonies, respectively, were picked out with sterilized toothpicks. The bacterial and actinomycete colonies were inoculated symmetrically and equidistantly at a distance of 2.5 cm from the *M. alpina* YW25 block on the plate, and cultured at 28°C in the dark for 5 days. The plate inoculated with *M. alpina* YW25 was used as the control. All processing settings were triplicated. Inhibition rate (%) = (colony radius of control group − colony radius of treatment group)/colony radius of control group × 100 (Cong et al., 2019). The inhibition of *M. alpina* YW25 by rhizosphere microorganisms was divided into four grades: − (no inhibition), + (inhibition rate < 30%), ++ (inhibition rate 30–60%), and +++ (inhibition rate > 60%).

### Experimental Design and Sample Collection

The PDA plugs containing mycelia of *M. alpina* YW25 and *F. oxysporum* YFW32 with a diameter of 5 mm were cultured for 7 days at 28°C in PDA liquid medium separately at 180 rpm. The mycelium was filtered using gauze and diluted with sterile water to prepare a 1.2 × 10^7^/ml spore suspension, which was used for pot inoculation of ginseng. Three treatment groups were established: single inoculation of *M. alpina* YW25 (MA), single inoculation with *F. oxysporum* YFW32 (FO), and inoculation with *M. alpina* YW25 and *F. oxysporum* YFW32 (MA_FO).

Potted soil (not autoclaved) contains 25.05 mg/kg nitrate nitrogen, 0.69 mg/kg ammonium nitrogen, 1.18 mg/kg available phosphorus, 292.25 mg/kg available potassium, total nitrogen 10.2 mg/g, total phosphorus 8.59 mg/g, total potassium 20 mg/g, and organic matter 0.35 g/g and the pH was 6.96. Three-year-old ginseng seedings were planted in each pot and inoculated by root irrigation. In MA and FO treatments, each pot (1.5 kg flower soil) was inoculated with 10 ml spore suspension. In MA_FO treatment, *M. alpina* YW25 and *F. oxysporum* YFW32 spore suspensions were inoculated with 5 ml each, and 10 ml sterile water was used as the control (CK). The setup for each treatment was repeated five times.

Ginseng was harvested after 70 days of pot planting. It was carefully uprooted and gently shaken to remove loosely adhered soil from the roots. Subsequently, all ginseng rhizosphere soil samples from the same treatment were mixed, and the rhizosphere soil sample of the treatment was formed. The rhizosphere soil samples were divided into two parts, and one of these was immediately stored in a −80°C refrigerator for the detection of soil microbial diversity. The other was air-dried indoors and stored at room temperature after filtering through a 2 mm sieve for determination of various soil physical and chemical properties. After washing and drying five ginseng plants in each treatment, the length and fresh weight of ginseng plants were measured by scale and balance. The ginseng plants were then divided into root and aboveground parts. After surface disinfection, the samples were quickly frozen in liquid nitrogen and then stored at −80°C for the detection of ginseng microbial diversity and plant defense enzymes.

### Measurement of Soil Physicochemical Properties and Plant Defense Enzymes

Soil pH was measured using a pH meter (S010, Horiba, Japan). Nitrate and ammonium nitrogen were determined by 2 mol/L KCl extraction spectrophotometry (Li et al., 2021b). Available phosphorus was determined by NaHCO_3_ extraction and molybdenum–antimony resistance spectrophotometry (Yuan et al., 2020). Kjeldahl was used to determine total nitrogen (Arunrat et al., 2022). Total phosphorus was determined by sodium hydroxide alkali fusion–molybdenum–antimony anti spectrophotometry (Liu et al., 2022). Total potassium and available potassium were determined by flame atomic absorption spectrophotometry (Li et al., 2021a), and organic matter was determined by the loss-of-burning method (Salehi et al., 2011). Soil urease activity was determined by indophenol colorimetry (Adetunji et al., 2021), and the activities of soil catalase, acid phosphatase, and sucrase were determined using kits (Suzhou Grace Biotechnology Co. Ltd.; Zhou et al., 2020).

Harvested fresh ginseng root tissue was used to detect plant defense enzymes. The activities of peroxidase (POD), polyphenol oxidase (PPO), lipoxygenase (LOX), and phenylalanine ammonia lyase (PAL) were determined using microplate kits (NO. G0107W, NO. G0113W, NO. G0906W, and NO. G0114W, respectively, Suzhou Grace Biotechnology Co. Ltd.; Cheng et al., 2020; Yang et al., 2020).

### High-Throughput Sequencing and Analysis of 16S rDNA and Internal Transcribed Spacer Regions

High-throughput Illumina sequencing was used to characterize the microbial community structure in the soil and plant samples (Majorbio Bio-Pharm Technology Co., Ltd., Shanghai, China). The V3-V4 regions of the soil bacterial 16S rRNA genes were amplified using the primers 338F (5′-ACTCCTACGGGAGGCAGCAG-3′) and 806R (5′-GGACTACHVGGGTWTCTAAT-3′; Xu et al., 2019). To assess the ginseng bacterial community, two sets of primers targeting the V3-V4 region of 16S rRNA gene were designed. The first-round reaction was amplified with primers 799F (5′-AACMGGATTAGATACCCKG-3′) and 1392R (5′-ACGGGCGGTGTGTRC-3′; Cui et al., 2020). The second-round reaction was amplified with primers 799F (5′-AACMGGATTAGATACCCKG-3′) and 1193R (5′-ACGTCATCCCCACCTTCC-3′; Bulgarelli et al., 2015). The ITS1F-ITS2R region of the ginseng fungal gene was amplified using the primers ITS1F (5′-CTTGGTCATTTAGAGGAAGTAA-3′) and ITS2R (5′-GCTGCGTTCTTCATCGATGC-3′; Sun et al., 2018). Specific primers with barcodes were synthesized according to the designated sequencing region, and then the samples were amplified using a thermocycler (GeneAmp^®^ 9700, ABI, United States). The raw reads were deposited into the NCBI sequence read archive (SRA) under the submission ID SUB10895992.^2^

Bacterial PCR reactions were performed in triplicate, with 4 μl 5× FastPfu Buffer, 2 μl 2.5 mM dNTPs, 0.8 μl 5 μM forward primer, 0.8 μl 5 μM reverse primer, 0.4 μl FastPfu Polymerase, 0.2 μl bovine serum albumin (BSA), and 10 ng template DNA in a 20 μl reaction volume. The thermal cycling conditions for prokaryotic 16S rRNA gene from soil bacteria fragment amplification were as follows: 3 min at 95°C, 30 cycles of 30 s at 95°C, 30 s at 55°C, 45 s at 72°C, and 10 min at 72°C. The 16S rRNA gene from ginseng bacterial fragments was amplified in two rounds, and the thermal cycling conditions of amplification were as follows: first round: 3 min at 95°C, 27 cycles of 30 s at 95°C, 30 s at 55°C, 45 s at 72°C; and 10 min at 72°C; second round: 3 min at 95°C, 13 cycles of 30 s at 95°C, 30 s at 55°C, 45 s at 72°C, and 10 min at 72°C. Fungal PCR reactions were performed in triplicate with 2 μl 10× rTaq Buffer, 2 μl 2.5 mM dNTPs, 0.8 μl 5 μM forward primer, 0.8 μl 5 μM reverse primer, 0.2 μl rTaq polymerase, 0.2 μl BSA, and 10 ng template DNA in a 20 μl reaction volume. The thermal cycling conditions for prokaryotic ITS gene fragment amplification were as follows: 3 min at 95°C, 30 cycles of 30 s at 95°C, 30 s at 55°C, 45 s at 72°C, and 10 min at 72°C. The PCR products were identified by 2% agarose gel electrophoresis, purified using an AxyPrep DNA gel extraction kit (Axygen, Corning, NY, United States), and quantified using a QuantiFluor™-ST Blue Fluorescence Quantification System (Promega).

The amplified sub-library was sequenced on an Illumina PE250 platform (Biozeron, Shanghai, China). The effective sequences of all samples were obtained according to the barcode, and Trimmomatic (version 0.36; Lohse et al., 2012) filtration was used to remove reads with an average mass of less than 20 in 50 bp. Sequences were assembled using FLASH with a minimum overlap of 10 bp and a maximum mismatch ratio of 0.2 (Magoc and Salzberg, 2011). The RDP classifier Bayesian algorithm (Wang et al., 2007; version 2.2) was used to classify the representative sequences of each operational taxonomic unit (OTU) with 97% similarity.^3^ The bacterial 16S rRNA comparison database was Silva (Release138; Quast et al., 2013)^4^ and the fungal ITS comparison database was Unite (Release 8.0; Koljalg et al., 2013).^5^

### Co-occurrence Network Analysis

A co-occurrence network based on the Spearman correlation coefficient matrix was constructed by NetworkX to study the relationship and interaction between bacteria and fungi in the aboveground and root of ginseng under different inoculation treatments. OTUs with relative abundance greater than 0.01% in each treatment were screened for OTU with subsequent correlation network construction. The most important interaction was highlighted, and the Spearman correlation threshold was set to 0.7, *p* < 0.05. Each node represents an OTU, and each edge represents a strong and significant correlation between the different nodes. Networks were visualized using the Gephi platform.^6^ Topological features (average degree and modularity) of the networks were calculated using NetworkX on the free online platform of Majorbio Cloud Platform.^7^

### Statistical Analysis

GraphPad Prism 8.3.0 was used to draw line and bar charts. SPSS 19.0 was used for one-way analysis of variance (ANOVA), and the significance level was *p* < 0.05. For the high-throughput Illumina sequencing data, Adonis test, Student’s *t*-test along with alpha diversity, db-RDA, and linear regression analyses were performed using the online platform of Majorbio Cloud.^8^

## Result

### Growth-Promoting Potential of *Mortierella alpina* YW25

**Figure 1:**
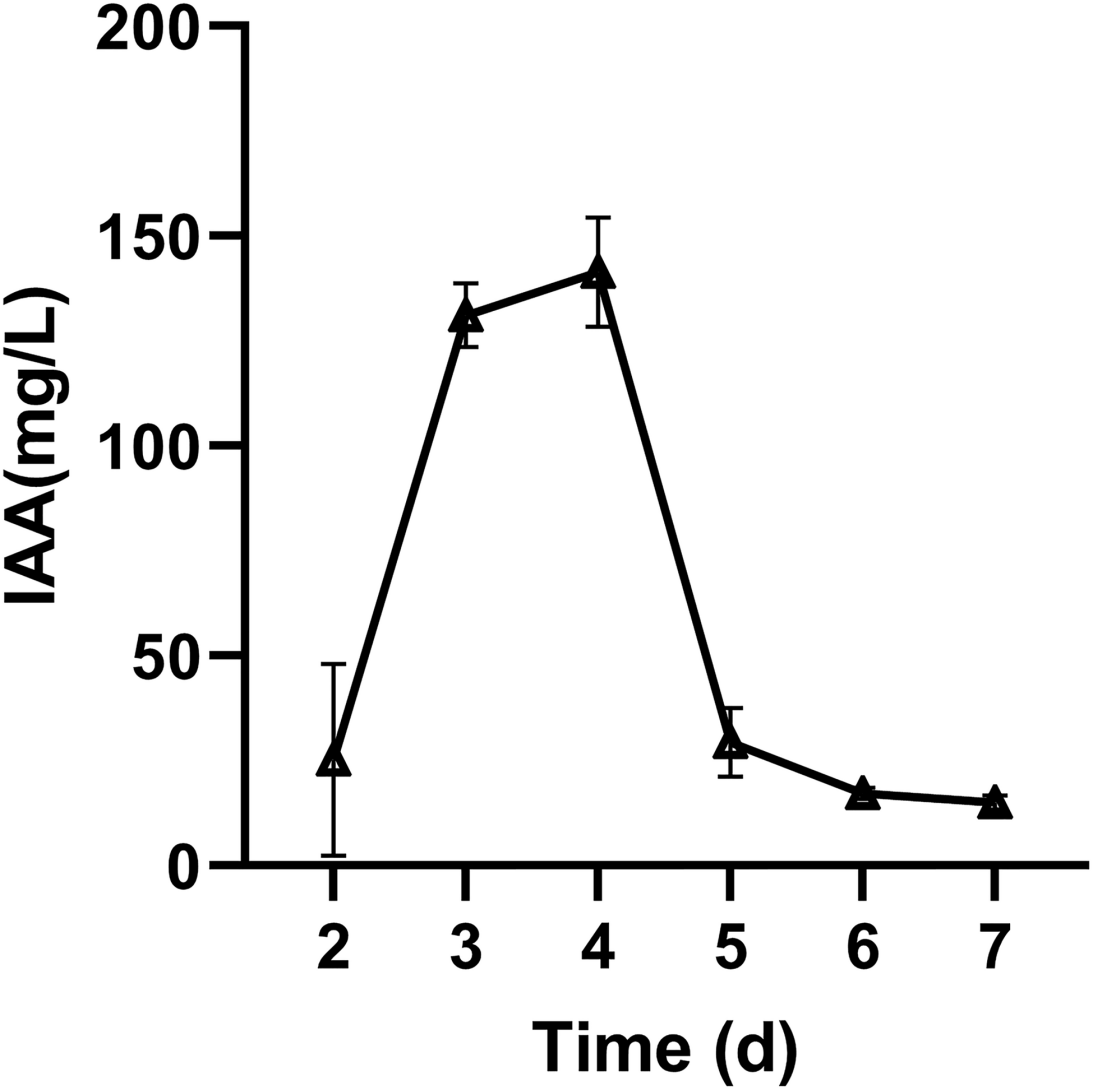
Effects of incubation time on the production of IAA by *Mortierella alpina* YW25.

To detect the plant growth-promoting potential of *M. alpina* YW25, the capacities of IAA and siderophore production, phosphorus solubilization, and hydrolase activity of *M. alpina* YW25 were determined. The results showed that it had a high ability to produce IAA, and the highest IAA concentration in PDB liquid medium containing 3 mM tryptophan reached 141.37 mg/L at 4 days (Figure 1). The ability to produce siderophores and dissolved phosphorus was not detected in *M. alpina* YW25. No hydrolase activities of *M. alpina* YW25, except for protease activity was 5.5 U/ml after 5 days.

### *In vitro* Analysis of *Mortierella alpina* YW25 and Rhizosphere Microbes Interactions

**Figure 2:**
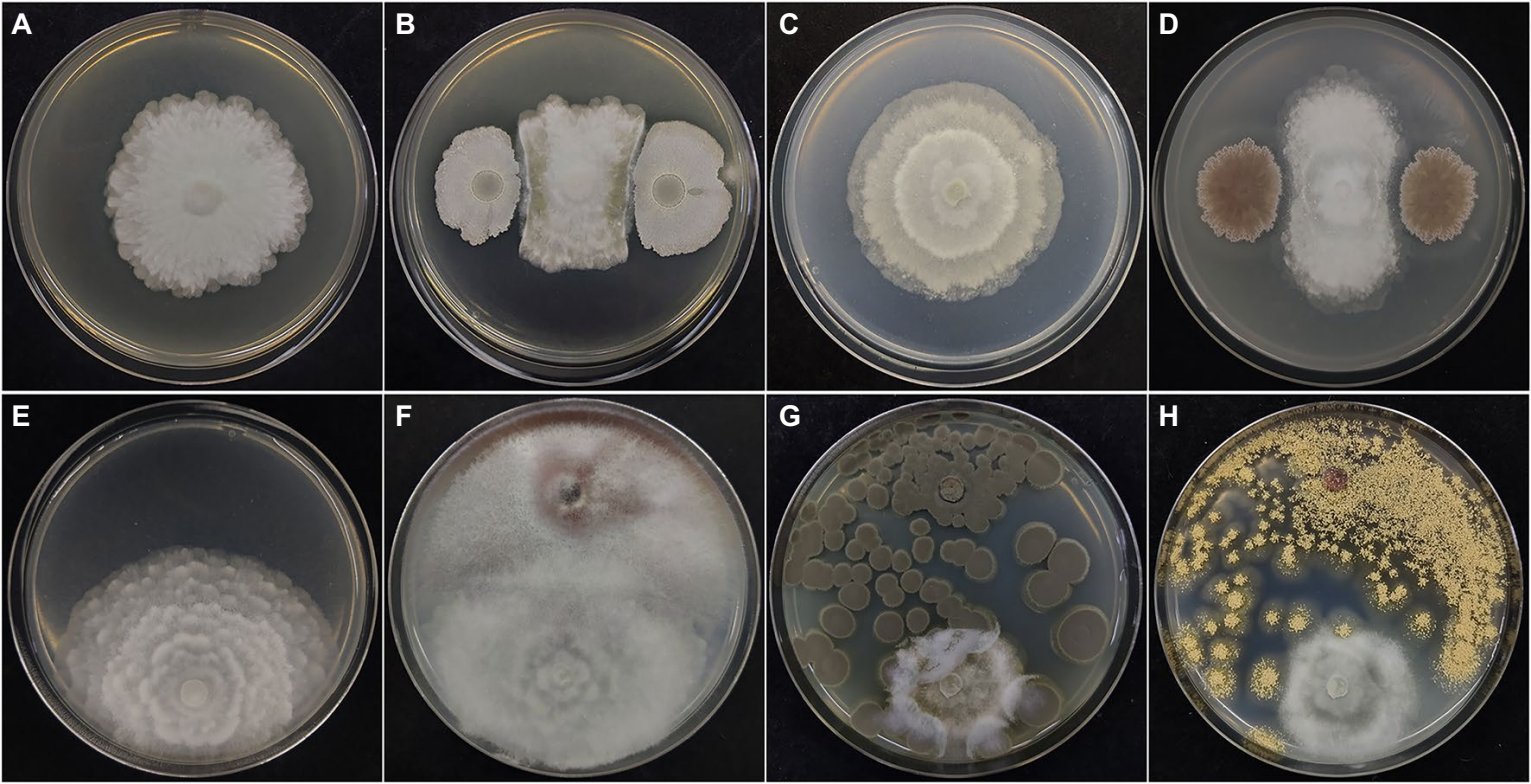
Results of confrontation between *Mortierella alpina* YW25 and rhizosphere microorganisms. As the control, *Mortierella alpina* YW25 was inoculated separately on LB **(A)**, Gao’s No.1 **(C)** medium for 5 days, and on PDA medium 7 days **(E)**. *Mortierella alpina* YW25 was co-cultured with *Bacillus velezensis* **(B)** on LB medium, *Streptomyces tricolor* **(D)** on Gao’s No.1 medium at 28°C for 5 days, and with *Fusarium oxysporum* YFW32 **(F)**, *Penicillium citrinum* **(G)**, *Aspergillus ochraceus* **(H)** on PDA medium at 28°C for 7 days.

The interaction between *M. alpina* YW25 and a variety of microorganisms in the ginseng rhizosphere were studied by plate confrontation experiments (Table S1 and Figure 2). Bacillus species (*Bacillus siamensis*, *B. velezensis*, *B. toyonensis*, *B. cereus*, and *B. zhangzhouensis*) significantly inhibited the growth of *M. alpina* YW25 among the 15 bacterial strains isolated from the ginseng rhizosphere. *Streptomyces tricolor* and *Brevibacterium frigoritolerans* (actinomycetes) also showed significant inhibition of *M. alpina* YW25. Fungi isolated from ginseng rhizosphere, such as *Trichoderma koningiopsis*, *T. viridescens*, *T. harzianum*, *T. velutinum*, *Rhizopus oryzae*, *Penicillium citrinum*, *P. chrysogenum*, *Aspergillus ochraceus*, *A. flavus*, *Cladosporium anthropophilum*, and *C. cladosporioides*, also inhibited the growth of *M. alpina* YW25. However, there was no obvious interaction between *F. oxysporum* YFW32 and *M. alpina* YW25.

### Effects of Inoculation With *Mortierella alpina* YW25 on *Panax ginseng*

**Figure 3:**
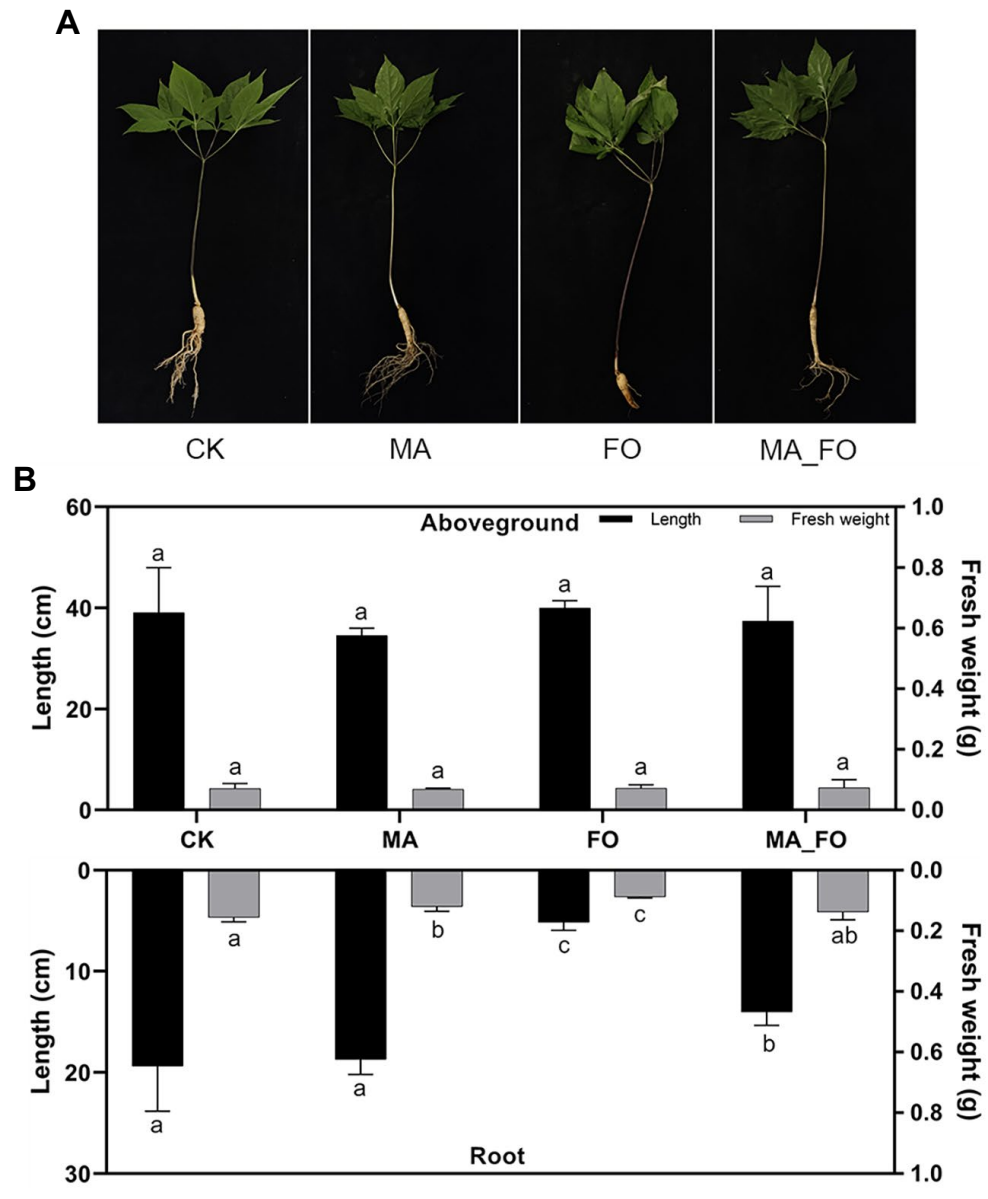
Growth status of ginseng under different inoculation treatments. **(A)** Photos of ginseng under different inoculation treatments. **(B)** The length and fresh weight of different parts of ginseng under different inoculation treatments. CK: control, FO: *Fusarium oxysporum* YFW32, MA: *Mortierella alpina* YW25, MA_FO: *Mortierella alpina* YW25 and *Fusarium oxysporum* YFW32, the same as below. The length and fresh weight of aboveground and root of ginseng under different treatments were analyzed for difference significance separately, and different lowercase letters indicated significant difference (*p* < 0.05).

To determine the effects of *M. alpina* YW25 inoculation on ginseng, plant height, root length, and fresh weight of both aboveground and root regions were measured (Figure 3). The defense enzymes of ginseng roots were also determined (Table 1). In the FO treatment, leaves withered and roots were infected and decomposed (Figure 3A). Ginseng in the other treatments showed no disease symptoms. The root length of FO was significantly lower than that of the other treatments (*p* < 0.05), and there was no significant difference between MA and CK. There was no significant difference in fresh weight of ginseng aboveground. The fresh weight of ginseng roots in FO was significantly lower than that in the other treatments. The fresh weight of ginseng roots in CK was significantly higher than that in MA, but there was no significant difference between CK and MA (Figure 3B). In the FO treatment, POD and PPO activities were 133.09 and 213.33, respectively, which were significantly higher than those in CK (*p* < 0.05). LOX activity was significantly lower, whereas PAL activity showed no significant change. Compared with CK, the activities of PPO, LOX, and PAL in the MA_FO were higher, but the activities of the four plant defense enzymes in the MA and MA_FO treatments were not significantly different (Table 1).

**Table 1:** Plant defense enzyme activities of ginseng root treated by differential inoculation.

	POD/U	PPO/U	LOX/U	PAL/U
CK	31.55 ± 5.31 b	107.73 ± 20.62 b	2093.3 ± 177.6 ab	30.06 ± 3.11 a
MA	29.97 ± 3.65 b	175.47 ± 7.74 ab	1386.0 ± 373.5 ab	34.40 ± 7.61 a
FO	133.09 ± 15.25 a	213.33 ± 30.08 a	1329.0 ± 174.2 b	31.94 ± 3.57 a
MA_FO	26.79 ± 0.96 b	162.27 ± 43.55 ab	2341.3 ± 676.3 a	37.73 ± 7.75 a

*POD, peroxidase; PPO, polyphenol oxidase; LOX, lipoxygenase; PAL, phenylalanine ammonia lyase. Different lowercase letters indicated significant difference (*p* < 0.05)*.

### Effects of *Mortierella alpina* YW25 Inoculation on Soil Physicochemical and Enzymatic Properties

Soil pH, ammonium nitrogen (NH_4_^+^-N), nitrate nitrogen (NO_3_^−^-N), total nitrogen (TN), available phosphorus (AP), total phosphorus (TP), available potassium (AK), total potassium (TK), urease (Urease), catalase (CAT), sucrase (SC), and acid phosphatase (ACP) were measured in ginseng roots (Table 2).

**Table 2:** Physicochemical and enzyme activity characteristics of ginseng rhizosphere soil under different inoculation treatments.

	CK	MA	FO	MA_FO
pH	6.56 ± 0.31 a	6.31 ± 0.02 ab	6.06 ± 0.04 b	6.37 ± 0.02 ab
NO_3_^−^-N/(mg/kg)	22.48 ± 0.06 a	17.58 ± 0.25 b	11.69 ± 1.79 c	4.48 ± 0.43 d
NH_4_^+^-N/(mg/kg)	0.38 ± 0.09 c	14.22 ± 1.51 a	2.38 ± 0.48 c	9.27 ± 1.58 b
AP/(mg/kg)	1.45 ± 0.11 b	1.99 ± 0.02 a	1.37 ± 0.04 b	1.08 ± 0.04 c
TN/(mg/g)	8.76 ± 0.15 ab	8.45 ± 0.12 b	8.83 ± 0.17 a	8.51 ± 0.28 ab
TP/(mg/g)	0.88 ± 0.01 a	0.84 ± 0.03 a	0.87 ± 0.04 a	0.87 ± 0.02 a
TK/(mg/g)	20.95 ± 0.18 a	19.95 ± 0.27 b	19.4 ± 0.16 c	19.68 ± 0.08 bc
AK/(mg/kg)	311.00 ± 1.87 c	346.75 ± 4.49 b	363.00 ± 2.12 a	351.00 ± 4.53 b
SOM(g/g)	0.22 ± 0.01 a	0.12 ± 0.07 a	0.14 ± 0.07 a	0.13 ± 0.01 a
Urease/(μg/g)	11.50 ± 0.72 b	14.55 ± 1.35 a	13.61 ± 1.26 ab	13.94 ± 0.42 a
CAT/(μmol/h/g)	293.06 ± 1.09 a	207.27 ± 2.29 c	285.34 ± 1.71 b	289.82 ± 0.60 a
SC/(mg/d/g)	41.11 ± 2.75 b	46.54 ± 0.65 a	35.48 ± 0.57 c	45.76 ± 2.83 ab
ACP/(μmol/h/g)	1.43 ± 0.01 bc	2.08 ± 0.19 a	1.59 ± 0.13 b	1.31 ± 0.03 c

*NO_3_^−^-N, nitrate nitrogen; NH_4_^+^-N, ammonium nitrogen; AP, available phosphorus; TN, total nitrogen; TP, total phosphorus; TK, total potassium; AK, available potassium; SOM, organic matter; Urease, urease activity; CAT, catalase activity; SC, sucrase activity; ACP, acid phosphatase activity; the same as below. Different lowercase letters indicated significant difference (*p* < 0.05)*.

As shown in the table, there were no significant differences in pH, TN, and TP among the MA, MA_FO, and CK treatments. The NO_3_^−^-N and TK content in MA rhizosphere soil was significantly lower than those in CK, and the NH_4_^+^-N, AP, and AK content was significantly higher than those in CK (*p* < 0.05). In the FO treatment, soil pH was significantly lower than that in CK, but the soil AK content was significantly higher than that of the other treatments (*p* < 0.05). The content of NO_3_^−^-N, AP, and AK in the MA_FO rhizosphere soil was significantly lower, but the content of NH_4_^+^-N was significantly higher than that in FO (*p* < 0.05). The available nitrogen (NH_4_^+^-N and NO_3_^−^-N) and AP content in the MA treatment was significantly higher than those in the MA_FO treatment. In addition, the activity of urease, SC, and ACP in MA soil was significantly higher, while the activity of CAT was significantly lower than that in CK (*p* < 0.05). The activities of CAT and SC in the MA_FO treatment were significantly higher, while the activity of ACP was significantly lower than that in FO.

### Effects of *Mortierella alpina* YW25 Inoculation on Plant and Soil Microbiome

**Figure 4:**
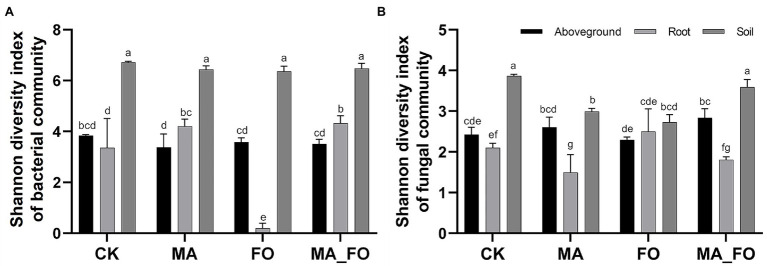
Shannon diversity index of bacterial **(A)** and fungi **(B)** community structure at different ecological niches (Aboveground: aboveground of ginseng; Root: root of ginseng; Soil: rhizosphere soil). Different lowercase letters indicate significant difference (*p* < 0.05).

The Shannon index was used to evaluate the soil microbial diversity of ginseng aboveground, roots, and rhizosphere under different treatments (Figure 4). The bacterial diversity in the ginseng rhizosphere soil was higher than that in the plant (*p* < 0.05) in all treatments. In the FO and MA treatments, soil fungal diversity was significantly lower than that in the CK and MA_FO treatments (*p* < 0.05). There was no significant difference between MA and CK in the diversity of bacteria and fungi in the aboveground parts of ginseng. In the MA treatment, the fungal diversity was significantly lower in the roots of ginseng than in CK and FO. Further, FO and MA_FO showed no significant differences in the diversity of bacteria in the aboveground parts of ginseng. Compared with FO, the bacterial diversity significantly increased, and the fungal diversity significantly decreased in the roots of ginseng in MA_FO (*p* < 0.05).

**Figure 5:**
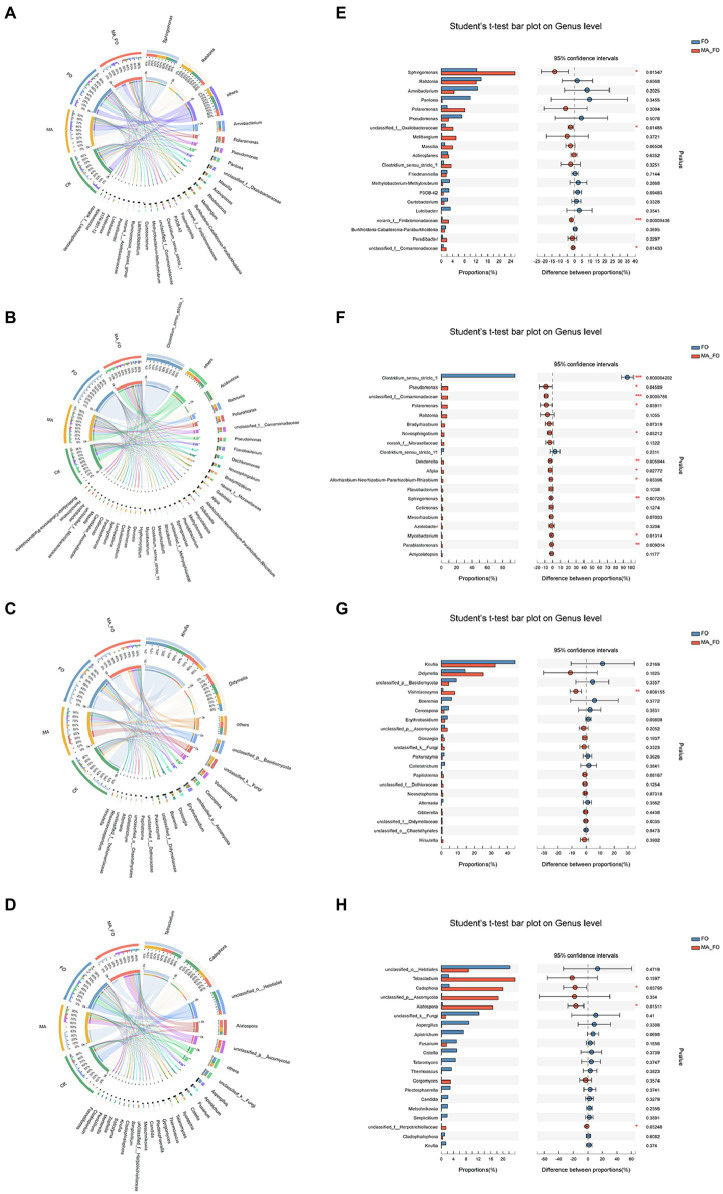
Analysis of microbial composition and differences in ginseng. Effects of different inoculation treatments on internal bacteria (**(A)** aboveground of ginseng; **(B)** root of ginseng) and fungi (**(C)** aboveground of ginseng; **(D)** root of ginseng) of ginseng. The left semicircle represents the different inoculation treatments. The right semicircle represents the dominant genera and proportions of each genus in different samples. Student’s *t*-test was used to test the significance of differences between FO and MA_FO at the genus level (**(E)** aboveground bacteria of ginseng; **(F)** root bacteria of ginseng; **(G)** aboveground fungi of ginseng; **(H)** root fungi of ginseng). The *y*-axis represents the species names at the genus level, the *x*-axis represents the average relative abundance in different groups of species, and the columns with different colors represents different groups. The far right is the value of *p*, *p < 0.05; **p < 0.01; ***p < 0.001.

There were also significant differences in the bacterial (Adonis, *R*^2^ = 0.2943, *p* = 0.001) and fungal (Adonis, *R*^2^ = 0.4483, *p* = 0.001) community structures in different parts of ginseng. Visual circos of microorganisms in aboveground and root of ginseng were constructed using bacteria and fungi genera, respectively, with relative abundance greater than 1% to evaluate the relationship between microorganisms and samples in different parts of ginseng (Figure 5).

The relationship between bacteria of aboveground ginseng parts and different treatments is shown in Figure 5A. *Sphingomonas*, *Ralstonia*, *Amnibacterium*, and *Polaromonas* were the main bacterial genera in the aboveground ginseng. The proportions of *Sphingomonas* and *Polaromonas* in MA_FO treatment were 34% and 51%, respectively, and *Ralstonia* had the highest distribution in MA treatment (41%). Compared with the FO treatment, the relative abundance of *Sphingomona*, Oxalobacteraceae, Fimbriimonadaceae, and Comamonadaceae in the MA_FO treatment was significantly increased (Figure 5B). *Clostridium* is the main bacterial genus of FO-treated ginseng roots and had a relative abundance of 96%. The main bacterial genera in CK roots were *Acidovorax*, *Flavobacterium*, and *Dechioromonas* with relative abundances of 28%, 9.9%, and 11%, respectively (Figure 5A). The bacterial diversity in ginseng roots between the MA and MA_FO treatments was significantly higher than that between the CK and FO treatments (*p* < 0.05), and there was no significant difference between the MA and MA_FO treatments. Compared to FO, the relative abundances of *Pseudomonas*, Comamonadaceae, *Polaromonas*, *Novosphingobium*, *Dokdonella*, *Afipia*, *Rhizobium*, *Sphingomonas*, *Mycobacterium*, and *Parablastomonas* increased significantly in ginseng roots after MA_FO treatment (Figure 5B).

*Knufia* and *Didymella* are the main fungal genera in the aboveground parts of ginseng. The relative abundance of *Knufia* in the MA treatment was 15%, which was significantly lower than that in the other treatments (*p* < 0.05; Figure 5A). In MA_FO, the species of potential plant pathogens in the aboveground parts of ginseng, such as *Didymella*, *Cercospora*, *Boeremia*, and *Alternaria*, were less than that of FO, and the relative abundance of *Vishniacozyma* was significantly higher than that of FO (*p* < 0.05; Figure 5B). Among the ginseng roots treated with CK, MA, and MA_FO, *Tetracladium*, Helotiales, *Cadophora*, and *Alatospora* were the main fungal genera. The relative abundances of Helotiales (1.3% and 8.8%) and *Cadophora* (22% and 20%) in MA and MA_FO were lower than those in CK, and *Tetracladium* (41% and 24%) and *Alatospora* (9.9% and 17%, respectively) were significantly higher than those in CK (Figure 5A). The relative abundance of *Cadophora* and *Alatospora* in the FO treatment was significantly lower than that in the MA_FO treatment (*p* < 0.05; Figure 5B). However, in terms of the distribution of fungi in different treatments, *Aspergillus*, *Plectosphaerella*, *Candida*, *Cladosporium*, and *Cladophialophora* had the highest distribution proportion in the FO treatment (89%, 100%, 83%, 91%, and 56%; Figure 5A).

**Figure 6:**
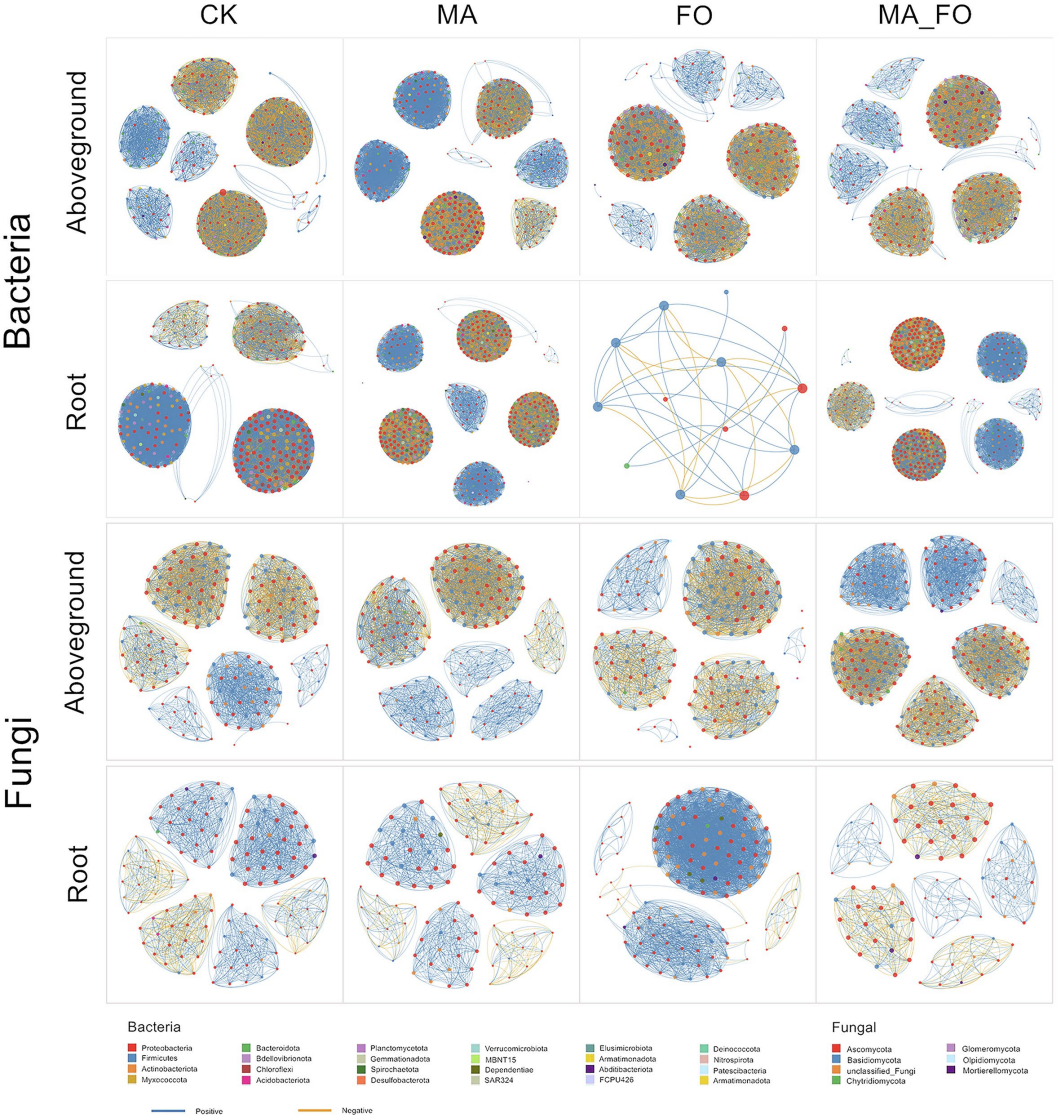
Analysis of microbial co-occurrence network in ginseng under different inoculation treatments. The nodes are colored according to bacterial and fungal phylum. Node size indicates the relative abundance of OTU. Edge color represents positive (blue) and negative (orange) correlations.

The co-occurrence networks of bacterial and fungal communities significantly varied in different parts of ginseng and among the different treatments (Figure 6, Tables 3 and 4). Except for FO treatment, the bacterial network structure of ginseng root was generally more complex (based on the number of edges and nodes, and average degree) than that of the aboveground parts of ginseng. Among all bacterial networks, the FO-treated bacterial network of ginseng root was the simplest (nodes: 13; edges: 32; average degree: 4.932). Compared with FO treatment, MA_FO had a higher proportion of negative correlation between aboveground and root bacterial networks (aboveground: 42.88%; root: 39.6%) and modularity (aboveground: 0.713; root: 0.646). The number of edges and nodes, average degree, and modularity of the bacterial networks in the aboveground and root of ginseng in MA were higher than those of the control, but the proportion of negative correlation was lower than that of the control. MA treatment had a more complex bacterial network (based on the number of edges and nodes, and average degree) than MA_FO, but MA_FO might have a more stable bacterial network (based on the negative correlation ratio).

**Table 3:** Key topological features of bacterial networks in aboveground and root of ginseng under different inoculation treatments.

	Aboveground				Root			
	CK	MA	FO	MA_FO	CK	MA	FO	MA_FO
Nodes	313	395	264	265	316	472	13	479
Edges	9,096	14,360	6,999	6,402	15,415	20,297	32	25,101
Positive edges ratio (%)	57.26	67.14	58.34	57.12	88.23	61.37	62.5	60.4
Negative edges ratio (%)	42.74	32.86	41.66	42.88	11.77	38.63	37.5	39.6
Average degree	58.121	72.709	53.023	48.317	97.563	86.004	4.923	104.806
Modularity	0.709	0.716	0.693	0.713	0.531	0.751	0.225	0.646

**Table 4:** Key topological features of fungal networks in aboveground and root of ginseng under different inoculation treatments.

	Aboveground	Root
	CK	MA	FO	MA_FO	CK	MA	FO	MA_FO
Nodes	175	174	143	243	120	110	130	86
Edges	2,917	2,984	2,228	5,428	1,442	1,028	3,056	628
Positive edges ratio (%)	64.38	65.45	58.44	68.24	86.62	86.19	99.08	63.06
Negative edges ratio (%)	35.62	34.55	41.56	31.76	13.38	13.81	0.92	36.94
Average degree	33.337	34.299	31.161	43.193	22.185	18.691	47.015	14.605
Modularity	0.742	0.687	0.681	0.794	0.783	0.79	0.342	0.772

The fungal network of ginseng is simpler since it has fewer nodes and edges compared to the bacterial network (Figure 6; Table 4). In contrast to the bacterial networks, the fungal network structure of the aboveground parts of ginseng is generally more complex than that of the root (based on the number of edges and nodes, and average degree); FO being the exception. The number of edges and nodes, average degree, and modularity of the fungal network in the aboveground parts of ginseng treated with MA_FO were higher than those in FO, while the opposite trend was observed in the root network of ginseng. However, the proportion of negative correlation was lower than that of FO. The fungal network of ginseng roots had a very high positive correlation ratio under the treatment of FO (99.08%). The number of edges and nodes, and average degree of the fungal networks in the aboveground ginseng treated with MA were higher than those of the control, but the proportion of negative correlation and modularity were lower than those of the control. The fungal networks of ginseng roots showed the opposite trend.

**Figure 7:**
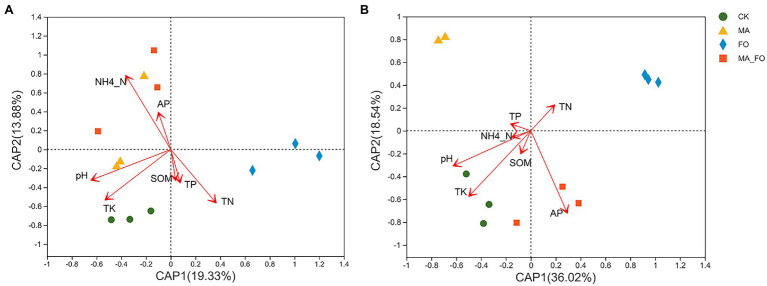
db-RDA analysis of soil microbial community and environmental factors. **(A)** bacterial community; **(B)** fungal community. Arrows represent environmental factors.

**Figure 8:**
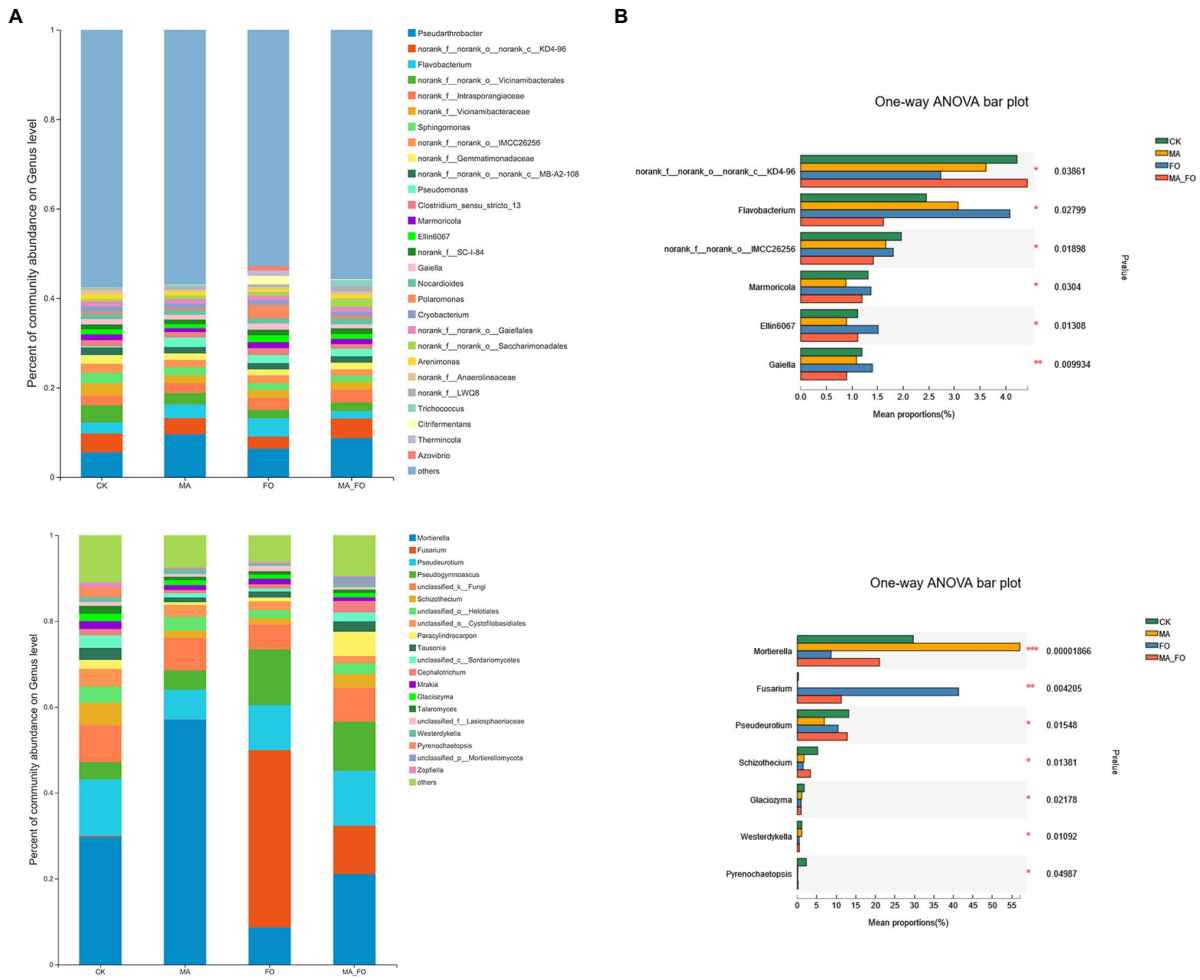
Analysis of microbial composition and differences in ginseng rhizosphere soil. **(A)** Relative abundance at the genus level of bacteria and fungi, where “others” represents species with relative abundance less than 1% in all samples; **(B)** One-way univariate analysis of variance (ANOVA) was used to test the significance of differences between groups at the genus level of bacteria and fungi. The y-axis represents the species names at the genus level, the *x*-axis represents the average relative abundance in different groups of species, and the columns with different colors represents different groups. Far right is the value of *p*, *p < 0.05; **p < 0.01; ***p < 0.001.

The bacterial community structure of ginseng rhizosphere soil under FO treatment was significantly different from that under the other treatments (Figure 7A). *Pseudarthrobacter* was the bacterium with the highest relative abundance in the rhizosphere soil of ginseng (5.5%–9.6%). The relative abundances of *Flavobacterium*, *Marmoricola*, *Gaiella*, and *Ellin6070* in FO were significantly higher than those in the other treatments (*p* < 0.05; Figure 8). There were no significant differences in soil fungal diversity and community structure between CK and MA_FO, but the diversity of FO and MA soils decreased significantly, and the fungal communities of FO and MA soil were significantly separated in CAP1 (36.02%). This suggests that FO and MA soils had different community structures (Figure 7B). *Fusarium* and *Mortierella* could colonize soil and become the dominant genera in FO and MA, with relative abundances of 41.3% and 57%, respectively. The relative abundance of *Fusarium* in MA_FO was significantly lower than that in FO, but the abundance of *Pseudeurotium* and *Schizothecium* was significantly higher (Figure 8).

### Correlation Between Soil Microorganisms and Physicochemical and Enzymatic Factors in Ginseng Rhizosphere

db-RDA and linear regression analyses were used to analyze the effects of soil physicochemical factors and enzyme activities on the soil microbial community structure. Using the variance inflation factor (VIF) to judge the collinearity between different soil physicochemical factors, the physicochemical factors with VIF > 10 in soil physical and chemical indicators were screened and removed. NO_3_^−^-N (VIF = 40.01) and AK (VIF = 72.16) were removed because they strongly correlated with other physicochemical factors.

The selected physicochemical factors were compared with soil bacteria (Figure 7A) and fungi (Figure 7B) for db-RDA analysis based on the Bray–Curtis distance. The results showed that pH (*r*^2^ = 0.5745, *p* = 0.011), NH_4_^+^-N (*r*^2^ = 0.9168, *p* = 0.001), TN (*r*^2^ = 0.5517, *p* = 0.032), and TK (*r*^2^ = 0.631, *p* = 0.023) were significantly correlated with bacterial community structure. Further, pH (*r*^2^ = 0.5775, *p* = 0.002), AP (*r*^2^ = 0.738, *p* = 0.007), and TK (*r*^2^ = 0.6945, *p* = 0.011) were significantly correlated with fungal community structure.

Linear regression analysis was used to evaluate the degree of explanation of the activity of sucrase, urease, ACP, and catalase to the variation in soil bacterial and fungal community structure (Supplementary Figure S1). Sucrase activity was significantly correlated with the community structure of soil bacteria (*R*^2^ = 0.5216, *p* = 0.008) and fungi (*R*^2^ = 0.4544, *p* = 0.0162). There was no significant correlation between urease, ACP, and catalase, and soil bacterial and fungal community structure.

## Discussion

### Growth-Promoting Characteristics of *Mortierella alpina* YW25 and Its *in vitro* Interaction With Rhizospheric Microorganisms

Plant-associated microbes are known to play important roles in plant health and disease (Kwak et al., 2018). Roots absorb water and inorganic nutrients from the soil and secrete organic exudates to shape the microbial diversity and structure of the soil (Bulgarelli et al., 2013). Exploring the interaction between plant and soil microbes and rhizospheric microorganisms is vital to prevent and suppress diseases, promote plant growth, or improve plant stress resistance (Mendes et al., 2013).

In this study, ginseng rhizosphere microorganisms were selected for the plate confrontation test to preliminarily study the interaction between *M. alpina* YW25 and the rhizosphere microorganisms. The results showed that the mycelial growth of *M. alpina* YW25 was inhibited by some probiotics in the rhizosphere, such as *Bacillus*, *Streptomyces*, *Brevibacterium*, *Trichoderma*, and *Penicillium* (Lee et al., 2021; Zhao et al., 2021a,b), as well as by some potential pathogens, such as *Cladosporium* and *Aspergillus* (Carolina Virginia et al., 2021; Tan et al., 2021). Antimicrobial substances (lipopeptides, antibiotics, and volatile organic compounds) secreted by *Bacillus* and metabolites of *Streptomyces* (e.g. quercetin) may inhibit the growth of *M. alpina* YW25 mycelia on plates (Awla and Rashid, 2020; Chen et al., 2020). However, it showed no sensitivity to other microorganisms, such as *Fusarium*, *Bjerkandera adusta*, *Trametes*, *Trichaptum abietinum* (Supplementary Table S1), and the soil-borne pathogen *F. oxysporum* during co-cultivation. The results of the pot experiment showed that *Mortierella* was negatively correlated with *Fusarium* and *Trichoderma* in ginseng rhizosphere soil (Spearman, −0.95 and −0.72). These negative correlations between *Mortierella* and *Fusarium* have also been observed in other systems (Hong et al., 2020; Xiang et al., 2021), which indicated that *Mortierella* did not directly inhibit the growth of *Fusarium*.

### Effects of Inoculation With Native *Mortierella alpina* YW25 on Physicochemical Properties of Ginseng Plants and Rhizosphere Soil

In this study, when *M. alpina* YW25 was singularly inoculated, the leaves of the ginseng plants expanded, fibrous roots developed, and it did not show any disease symptoms. There was no significant difference between the aboveground and root lengths of ginseng compared with CK (Figure 3B). This indicates that inoculation of *M. alpina* YW25 in ginseng rhizosphere did not result in ginseng root disease. We evaluated the growth-promoting characteristics of *M. alpina* YW25 and found that it had a high IAA production capacity, with a maximum value of 141.37 mg/L, which was much higher than the IAA yield of reported strains (Bader et al., 2020; Galeano et al., 2021). However, no obvious growth-promoting effect was observed in the ginseng plants. A study involving *M. capitata* inoculation showed that it could increase maize biomass and promote plant growth (Li et al., 2020a). This difference may be attributed to the different microbial or plant species in this study.

Interactions with microbial species and network modularity affect the community stability (Coyte et al., 2015). Compared with CK, MA significantly increased the diversity of root bacteria and significantly decreased the diversity of root fungi, but there was no significant difference in the microbial diversity of the aboveground parts of ginseng (Figure 4). In the co-occurrence network (Figure 6), MA was more complex than CK. Compared with CK, ginseng roots in MA had more edges and nodes in the bacterial network and fewer edges and nodes in the fungal network. Moreover, in the MA treatment, the ginseng root microbial network had a higher negative correlation ratio and modularity. The results showed that inoculation of *M. alpina* YW25 could increase the complexity of the bacterial community structure in ginseng root, while reducing the complexity and improving the stability of the ginseng root fungal community.

The results of this study showed that a single inoculation of *M. alpina* YW25 had significant effects on some nutrient content in ginseng rhizosphere soil. Phosphorus can enhance drought and disease resistance in plants and promote their growth and development. A lack of phosphorus leads to a significant decrease in crop yield (Elhaissoufi et al., 2021). The results of this study showed that the AP content and ACP activity of ginseng rhizosphere soil treated with MA were significantly higher than those in other treatments. Hence, inoculation with *Mortierella* increased the AP content in soil (Spearman, 0.70). This was the same as observed in previous studies (Li et al., 2020a; Guo et al., 2021), and indicated that *Mortierella* could dissolve inorganic phosphorus in soil. In addition, oxalates are also synthesized and released to help plants or mycorrhizal fungi obtain phosphorus (Qiang et al., 2021). However, *M. alpina* YW25 did not show phosphorus solubility in the PVK plate. The difference between plate cultivation and pot experimentation might be because dissolving phosphorus in the pot experiment was realized by regulating rhizosphere microorganisms. Compared with CK, Actinobacteria (*Pseudarthrobacter*, *Microbacterium*, and *Microlunatus*) and *Rhizobium* were significantly enriched in MA (Supplementary Figure S2). These microorganisms have their own biophosphorus conversion activity (Pindi, 2012). In addition, the content of soil available nitrogen (NH_4_^+^-N and NO_3_^−^-N) after *M. alpina* YW25 inoculation was significantly higher than that in FO. The AP content in the rhizosphere soil was positively correlated with the available nitrogen content (NH_4_^+^-N and NO_3_^−^-N; Spearman, 0.26 and 0.62), but negatively correlated with the available potassium content (Spearman, −0.36). The results showed that nitrogen and phosphorus availabilities were driven by each other between plants and soil (Xu et al., 2020). Phosphorus in soil also increases the retention of nitrogen in soil–plant systems, thereby reducing nitrogen loss due to soil leaching (Mehnaz et al., 2019).

### *Mortierella alpina* YW25 Could Aid in Plant Resistance Against Pathogenic Invasion After MA_FO Treatment

In FO treatment, ginseng plant showed the typical characteristics of root rot disease with wilted leaves, brown and rotten root (Punja et al., 2008). However, in the MA_FO treatment, the leaves of ginseng expanded and the roots did not show browning symptoms. This indicates that the treatment with MA_FO in ginseng rhizosphere could effectively resist root rot caused by *F. oxysporum* YFW32. Plant defense enzymes (POD, PPO, and PAL) in ginseng roots were detected while exploring the reason for disease resistance (Table 1), but these results are different from those of previous studies (Nandhini et al., 2018). Pattern recognition receptors located on the surface of plants can recognize microbe- or pathogen-associated molecular patterns, and this recognition can then stimulate cascade defense signals resulting in induced systemic resistance (ISR) in plants (Bukhat et al., 2020). ISR is associated with defense enzymes, such as POD, PPO, and PAL. When plants are under biotic stress, these enzymes are induced to help resist pathogens (Appu et al., 2021). Invasion of *F. oxysporum* YFW32 significantly increased POD and PPO activities in ginseng roots. However, the activities of these enzymes were not significantly increased in MA_FO, which suggests that *M. alpina* YW25 may not induce plant resistance of ginseng to resist pathogen invasion.

Furthermore, we investigated the plant-associated microbiomes. The structure of the plant microbiome is influenced by complex interactions between the host, microorganisms, and related environmental factors, such as climate, soil, and cultivation practices (Kmgd et al., 2020). Treatment with MA_FO had a significant effect on the ginseng microbiome (Figure 5). Compared with FO, treatment with MA_FO significantly increased bacterial diversity and decreased fungal diversity in ginseng roots. The relative abundances of *Pseudomonas*, Comamonadaceae (e.g., *Polaromonas*), Sphingomonadaceae (*Sphingomonas* and *Novosphingobium*), and *Rhizobium* in MA_FO were significantly higher than those in FO (Figure 5B). This result is similar to that of previous research on American ginseng with *Trichoderma atroviride* inoculation (Li et al., 2022). These root microbiotas showed high antagonistic ability against root-associated fungi (Duran et al., 2018). In addition, the relative abundance of some potential plant growth-promoting microorganisms, such as *Vishniacozyma*, *Cadophora*, and *Alatospora*, was higher in the MA_FO treatment than in FO (Bizabani and Dames, 2015; Artigas et al., 2017; Lutz et al., 2020). Therefore, we speculated that *M. alpina* YW25 may enrich plant growth-promoting microorganisms by stimulating ginseng plants, and absorbing more nutrients for plant growth while inhibiting the invasion and proliferation of potential pathogens.

The plant microbial community structure of ginseng treated with MA_FO was different from that treated with FO, and the effect of MA_FO treatment on root microbial community was greater than that of aboveground ginseng (Figure 6). In the ginseng root, the number of nodes and edges of the bacterial network in FO was much lower than that in the control, and the number of nodes and edges in the fungal network was higher than that in CK. This indicated that FO reduced the complexity of the bacterial network, but increased the complexity of the fungal network in the ginseng root. The same results were observed in co-occurrence networks of peppers infected with *Fusarium* wilt disease (Gao et al., 2021). The complexity of microbial networks may be related to alpha diversity (Fan et al., 2018). The modularity and negative correlation of the bacterial and fungal networks of ginseng roots treated with FO were also much lower than those of the control. Low modularity and negative correlation may increase the unstable effect of the community (Grilli et al., 2016; Hernandez et al., 2021). Compared with FO, MA_FO increased the complexity of the root bacterial network, reduced the complexity of the root fungal network (based on the number of edges, nodes, and average degree), and improved the stability of the root microbial community (based on modularity and negative correlation ratio).

In this study, we found that ginseng rhizosphere-inoculated fungi had an effect on soil properties and the rhizosphere soil microbial community (Table 2 and Figure 8). First, pH was significantly correlated with the changes in rhizosphere soil bacterial and fungal communities (*R*^2^ = 0.5745 and 0.5775, respectively; Figure 7), which was the main factor affecting soil microbial diversity and community structure (Kang et al., 2021). Second, soil NH_4_^+^-N content had the strongest correlation with soil bacterial community structure (*R*^2^ = 0.9168; Figure 7A), and the soil physical and chemical factors had the greatest influence on the composition of the rhizosphere bacterial community (Liu et al., 2020a). Compared with the FO treatment, the activities of NH_4_^+^-N, sucrase, and catalase in soil increased significantly in the MA_FO treatment. This suggested that when treated in MA_FO, MA may help improve soil fertility, provide more nutrients for plants and soil microorganisms, and bioremediate the soil (Stepniewska et al., 2009; Sellami et al., 2022).

The fungal diversity of the rhizosphere soil in MA_FO was significantly higher than that in the FO treatment, but there was no significant change in bacterial diversity (Figure 4). *Mortierella* inoculation with *Fusarium* significantly reduced the relative abundance of *Fusarium* in soil (Figure 8B), and there was no significant difference in the soil fungal community structure between the two treatments (Figure 7B). Previous studies have also shown that the abundance of *Mortierella* in soil was significantly negatively correlated with diseases in plants, such as apple (Wang et al., 2018), vanilla (Xiong et al., 2017), eggplant (Ogundeji et al., 2021), celery, and watermelon (Liu et al., 2020a). In conclusion, inoculation with *M. alpina* YW25 significantly inhibited the proliferation of *F. oxysporum* in ginseng rhizosphere soil but did not affect the health of the rhizosphere soil.

To conclude, *M. alpina* YW25 had the maximum yield of IAA at 4 days (141.37 mg/L). Inoculation of *M. alpina* in ginseng rhizosphere significantly alleviated the pathogenicity of *F. oxysporum* in ginseng plants, increased the content of available nitrogen and phosphorus in rhizosphere soil, and improved the activities of soil sucrase and ACP. *M. alpina* inoculation with *F. oxysporum* had the greatest effect on the microbial community in the ginseng roots and had a greater effect on the fungal community than on the bacterial community. *M. alpina* inoculation helped ginseng recruit more plant growth-promoting microorganisms, change the microbial structure of ginseng roots, and build a more stable microbial network of ginseng roots. Thus, it inhibited potential pathogens, effectively prevented the invasion of pathogens, and ensured healthy plant growth. Therefore, *M. alpina* helped *Panax ginseng* resist *F. oxysporum* infection by mainly regulating the fungal community in the root.

## Data Availability Statement

The datasets presented in this study can be found in online repositories. The names of the repository/repositories and accession number(s) can be found at: https://www.ncbi.nlm.nih.gov/, Biosample No. SAMN24474502.

## Author Contributions

YW and HY conceived and designed the experiment. YW and LW performed the experiment. YW, LW, and MS analyzed the data. YW wrote the paper. HW, MZ, and HY guided the research work and thoroughly reviewed and corrected English language of the manuscript. All authors contributed to the article and approved the submitted version.

## Funding

This study was supported by Fundamental Research Funds for the Central Universities (No. 2572020DR08 and No. 2572020DP07).

## Conflict of Interest

The authors declare that the research was conducted in the absence of any commercial or financial relationships that could be construed as a potential conflict of interest.

## Publisher’s Note

All claims expressed in this article are solely those of the authors and do not necessarily represent those of their affiliated organizations, or those of the publisher, the editors and the reviewers. Any product that may be evaluated in this article, or claim that may be made by its manufacturer, is not guaranteed or endorsed by the publisher.

## Footnotes

^1^ https://dfast.nig.ac.jp/

^2^ https://submit.ncbi.nlm.nih.gov/subs/sra/SUB10895992/overview

^3^ http://sourceforge.net/projects/rdpclassifier/

^4^ http://www.arb-silva.de

^5^ http://unite.ut.ee/index.php

^6^ http://gephi.github.io/

^7^ http://www.majorbio.com

^8^ https://cloud.majorbio.com/
