# Supplementary material for: Regulating Root Fungal Community Using Mortierella alpina for Fusarium oxysporum Resistance in Panax ginseng
Source: Front Microbiol. 2022 May 12;13:850917. doi: 10.3389/fmicb.2022.850917 (PMC9133625; doi:10.3389/fmicb.2022.850917)
Supplement: Supplementary file 1 [file Table_1.DOCX]

Table S1 Interaction between *Mortierella alpina* YW25 and rhizospheric microorganisms of ginseng.

| Bacteria & Actinomyces | |  | Fungi | |
| --- | --- | --- | --- | --- |
| Antagonistic microbe | Inhibitory effect |  | Antagonistic microbe | Inhibitory effect |
| *Bacillus siamensis* | ++ |  | *Rhizopus oryzae* | +++ |
| *Bacillus velezensis* | ++ |  | *Trichoderma koningiopsis* | +++ |
| *Bacillus toyonensis* | + |  | *Penicillium citrinum* | +++ |
| *Bacillus cereus* | + |  | *Aspergillus ochraceus* | +++ |
| *Bacillus zhangzhouensis* | + |  | *Trichoderma viridescens* | +++ |
| *Bacillus aryabhattai* | - |  | *Trichoderma harzianum* | +++ |
| *Bacillus altitudinis* | - |  | *Trichoderma velutinum* | +++ |
| *Bacillus megaterium* | - |  | *Cladosporium anthropophilum* | +++ |
| *Pseudomonas putida* | - |  | *Aspergillus flavus* | ++ |
| *Pseudomonas glycinae* | - |  | *Penicillium chrysogenum* | + |
| *Brevibacillus schisleri* | - |  | *Cladosporium cladosporioides* | + |
| *Enterobacter kobei* | - |  | *Fusarium oxysporum* | - |
| *Burkholderia ambifaria* | - |  | *Fusarium solani* | - |
| *Stenotrophomonas maltophilia* | - |  | *Bjerkandera adusta* | - |
| *Xanthomonas maliensis* | - |  | *Trametes velutina* | - |
| *Streptomyces tricolor* | ++ |  | *Trametes versicolor* | - |
| *Brevibacterium frigoritolerans* | ++ |  | *Trichaptum abietinum* | - |

Note: - (no inhibition)，+ (inhibition rate < 30%)，++ (inhibition rate 30–60%)，+++ (inhibition rate > 60%.
