# Supplementary material for: Regulating Root Fungal Community Using Mortierella alpina for Fusarium oxysporum Resistance in Panax ginseng
Source: Front Microbiol. 2022 May 12;13:850917. doi: 10.3389/fmicb.2022.850917 (PMC9133625; doi:10.3389/fmicb.2022.850917)
Supplement: Supplementary file 2 [file Table_2.DOCX]

Fig. S1. Linear regression analysis for evaluating correlation between the activities of invertase (SC), catalase (CAT), acid phosphatase (ACP), and urease (Urease) and the changes of soil bacterial (A–D) and fungal (E–H) community structure at genus level.

Fig. S2. Student’s t-test was used to test the significance of differences between CK and MA at the genus level of bacteria (A) and fungi (B) in ginseng rhizosphere soil. The Y-axis represents the species names at the genus level, the X-axis represents the average relative abundance in different groups of species, and the columns with different colors represent different groups. The far right is the P value, **p* < 0.05; ***p* < 0.01; ****p* < 0.001.
