# Supplementary figures and images for: Regulating Root Fungal Community Using Mortierella alpina for Fusarium oxysporum Resistance in Panax ginseng
Source: Front Microbiol. 2022 May 12;13:850917. doi: 10.3389/fmicb.2022.850917 (PMC9133625; doi:10.3389/fmicb.2022.850917)

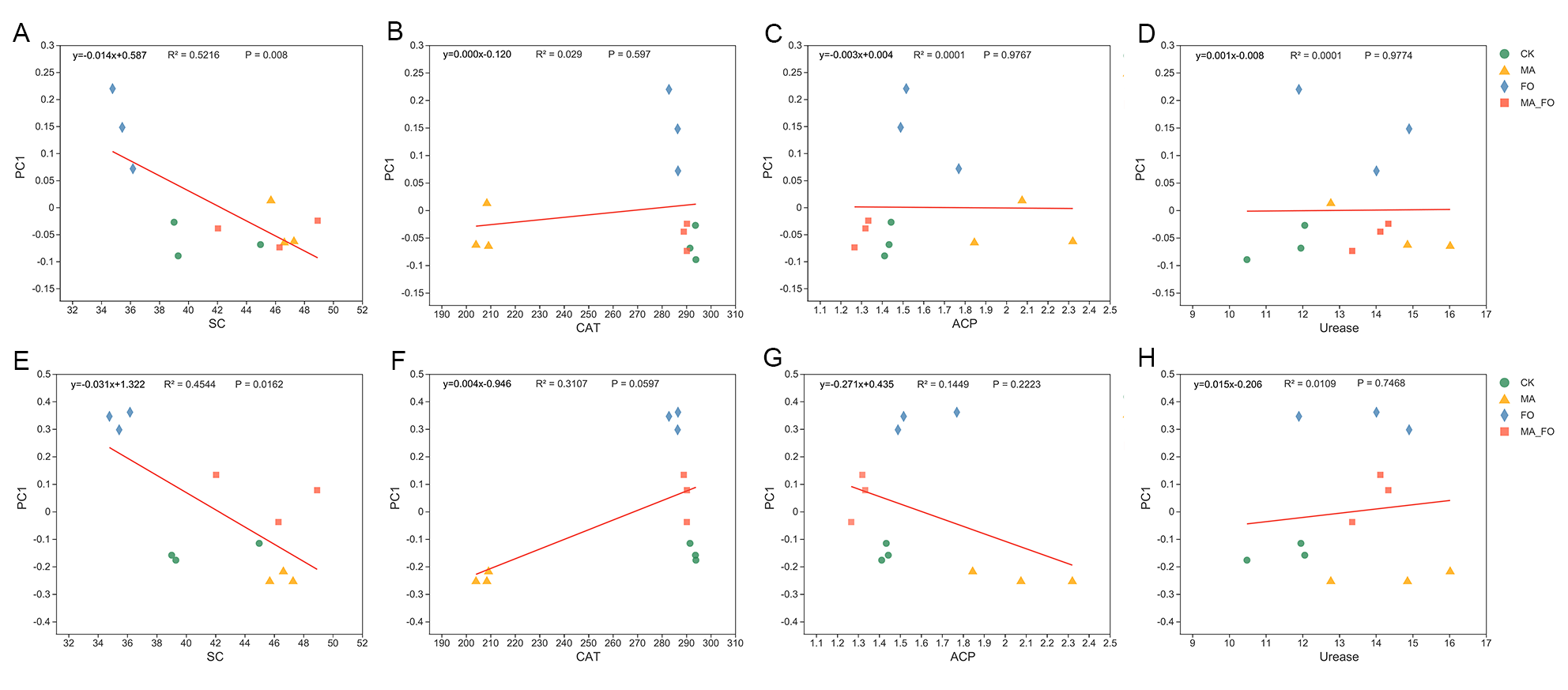

Supplement: Supplementary file 3 [file Image_1.TIF]

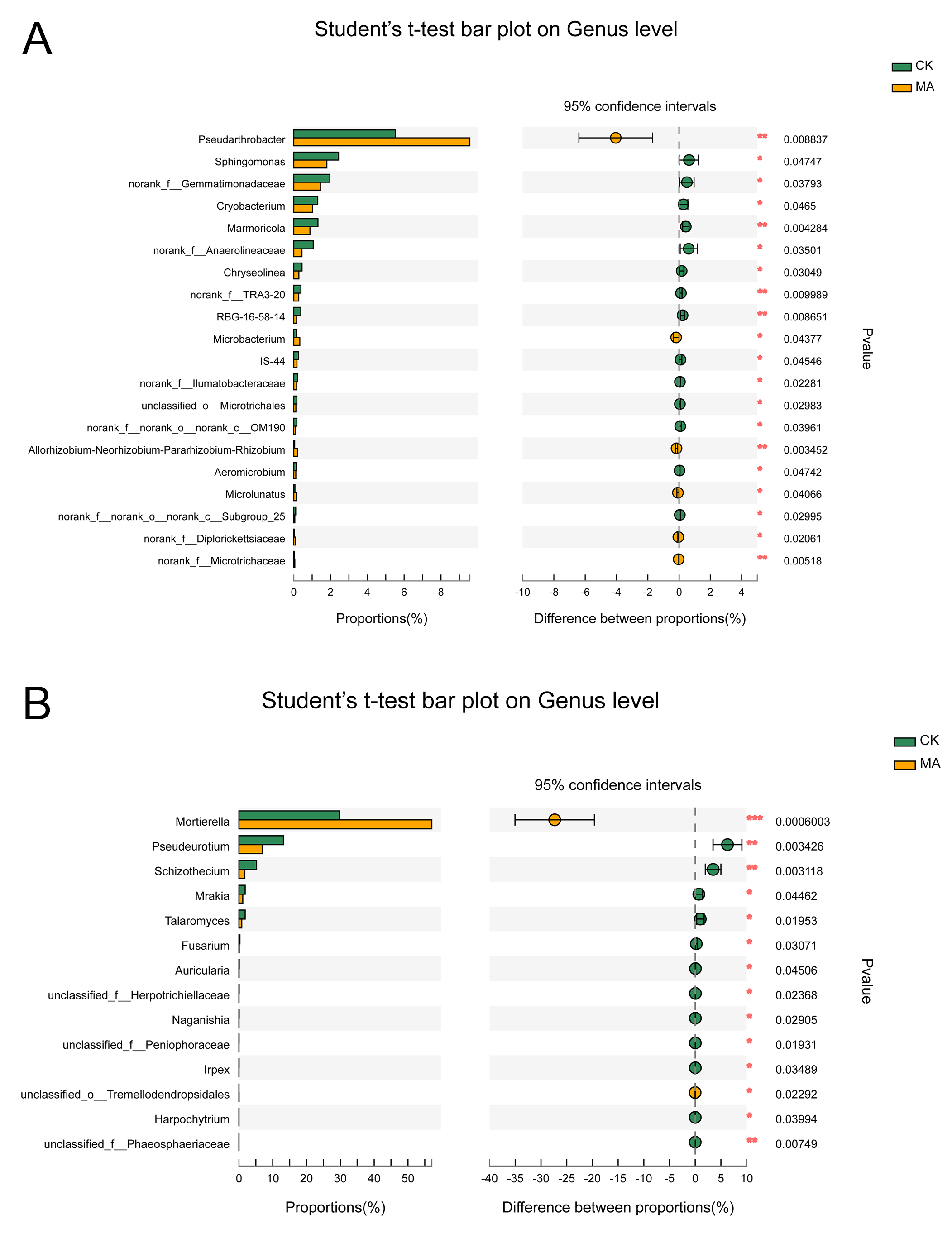

Supplement: Supplementary file 4 [file Image_2.TIF]
